# Effect of Alternate Treatment with Intravitreal Corticosteroid and Anti-VEGF for Macular Edema Secondary to Retinal Vein Occlusion

**DOI:** 10.1155/2021/5948113

**Published:** 2021-09-28

**Authors:** Young Hwan Bae, Seong Mi Kim, Jin Young Kim, So Hyun Bae, Hakyoung Kim, Dae Joong Ma

**Affiliations:** ^1^Department of Ophthalmology, Hallym University Kangnam Sacred Heart Hospital, Seoul, Republic of Korea; ^2^Department of Ophthalmology, Jeju National University College of Medicine, Jeju-si, Jeju-do, Republic of Korea

## Abstract

**Purpose:**

To evaluate whether treatment with intravitreal corticosteroid and anti-vascular endothelial growth factor (VEGF) injections alternately can improve treatment outcomes of macular edema (ME) caused by retinal vein occlusion (RVO).

**Methods:**

This dual-center retrospective study included 112 eyes with treatment-naïve ME secondary to RVO that were alternately treated with intravitreal corticosteroid and anti-VEGF injections (33 eyes, alternate group) or treated only with intravitreal anti-VEGF injections (79 eyes, anti-VEGF group) on a pro re nata basis.

**Results:**

During the 12-month follow-up period, the alternate group achieved a visual acuity gain of 0.39 logMAR, while the anti-VEGF group achieved a gain of 0.21 logMAR (*P*=0.042). The alternate group demonstrated a reduction in the central macular thickness of 229.9-*μ*m, while the anti-VEGF group achieved a reduction of 220.1 *μ*m (*P*=0.887). The alternate group required an average of 5.2 injections, while the anti-VEGF received 4.2 injections (*P* < 0.001). In a propensity score-matched cohort to compensate for the differences in the injection numbers between the two groups, the alternate group achieved a better visual acuity gain than the anti-VEGF group at month 12 (0.39 logMAR vs. 0.17 logMAR, *P*=0.048).

**Conclusions:**

In ME secondary to RVO, treatment with intravitreal corticosteroid and anti-VEGF injections alternately resulted in a more favorable visual outcome compared with intravitreal anti-VEGF monotherapy.

## 1. Introduction

Retinal vein occlusion (RVO) is the second most common retinal vascular disorder following diabetic retinopathy [1, 2]. It is characterized by engorgement and dilatation of the retinal veins due to increased retinal venous blood pressure, followed by the partial or complete obstruction of one or more retinal veins [3, 4]. RVO is associated with several complications, including macular edema (ME), vitreous hemorrhage, optic neuropathy, macular ischemia, or even tractional retinal detachment, which can result in visual loss [5].

Among these, ME is the most common complication of RVO that can lead to loss of vision. Multiple therapeutic options are used to treat ME secondary to RVO. Laser photocoagulation therapy was previously considered a treatment of choice for ME secondary to RVO [6, 7]. In recent times, intravitreal anti-vascular endothelial growth factor (VEGF) has become the first line of treatment [8]. However, a significant proportion of patients show an inadequate response to anti-VEGF therapy [9, 10]; hence, an alternative approach for the suboptimal responders is necessary.

Corticosteroids can effectively treat ME secondary to RVO due to their anti-inflammatory, antiangiogenic, and blood-retinal barrier-stabilizing properties [11]. Many therapeutic regimens incorporate corticosteroids, including their use as the first-line treatment [12], switching from anti-VEGF therapy in suboptimal responders [13] and using in combination with anti-VEGF agent [14].

The combination of corticosteroids and anti-VEGF agents might have synergistic effects in the treatment of ME secondary to RVO because the upregulation and pathological changes associated with the action of several inflammatory and vasogenic mediators are responsible for the development of ME secondary to RVO [15, 16]. Our previous work showed that a novel combination strategy that used intravitreal corticosteroid and anti-VEGF injections alternately resulted in a better visual outcome in patients with treatment-naïve diabetic macular edema (DME) than in those treated only with intravitreal anti-VEGF injections [17]. However, this novel treatment strategy has not been used in ME associated with other conditions.

This study assessed the efficacy of alternate treatment with intravitreal corticosteroid and anti-VEGF injections in improving the visual and anatomical outcomes of patients with ME secondary to RVO in a real-life setting.

## 2. Materials and Methods

### 2.1. Study Design

This dual-center retrospective study was conducted at Hallym University Kangnam Sacred Heart Hospital and Jeju National University Hospital in the Republic of Korea. The Institutional Review Board of Hallym University Kangnam Sacred Heart Hospital and Jeju National University Hospital approved the retrospective study protocol (IRB approval number: 2020-11-005 and 2019-10-003, respectively). This study was conducted in accordance with the tenets of the Declaration of Helsinki. The study included consecutive treatment-naïve eyes that received intravitreal injections between April 2009 and May 2020 for the treatment of ME secondary to RVO at both institutions.

A complete ophthalmological examination, which included best-corrected visual acuity (BCVA) measurement using the Snellen chart, tonometry, slit-lamp biomicroscopy, fundus examination, and optical coherence tomography (OCT) (DRI OCT Triton; Topcon, Tokyo, Japan or Cirrus HD-OCT; Carl Zeiss Meditec, Dublin, CA, USA), was performed at the initial visit and was repeated at every follow-up visit for all patients. The central macular thickness (CMT) was obtained from the central 1 mm subfield area from the OCT images.

The exclusion criteria were as follows: (1) CMT that was 250 *μ*m or less on OCT; (2) prior history of intravitreal injections of anti-VEGF or corticosteroids; (3) prior intraocular surgeries except for cataract surgery; (4) ocular conditions other than RVO that might affect the macula (e.g., age-related macular degeneration, diabetic retinopathy more severe than a mild nonproliferative disease, vitreomacular traction, epiretinal membrane, macular telangiectasia, or uveitis); (5) uncontrolled ocular hypertension (defined as intraocular pressure (IOP) ≥25 mmHg despite antiglaucoma therapy); (6) follow-up period shorter than 12 months; and (7) intravitreal injections less than thrice during the follow-up period.

In this real-life study, all patients received an intravitreal injection at baseline and were followed up almost every month and treated on a pro re nata (PRN) basis, based on the OCT findings. Retreatment was typically performed in cases with a recurrence of ME, defined as the presence of intraretinal cysts on OCT images and associated with visual impairment. The drug for intravitreal injection, e.g., 1.25 mg/0.05 mL bevacizumab (Avastin; Genentech/Roche, South San Francisco, California, USA), 0.5 mg/0.05 mL ranibizumab (Lucentis; Genentech/Roche), 2.0 mg/0.05 mL aflibercept (Eylea; Regeneron, Tarrytown, New York, USA), 4 mg/0.1 mL triamcinolone acetonide (Triam; Shin-Poong Pharmaceutical Co. Ltd., Seoul, Korea), or 0.7 mg sustained-release dexamethasone implant (Ozurdex, Allergan, Inc., Irvine, CA, USA), was at the treating physician's discretion.

**Figure 1:**
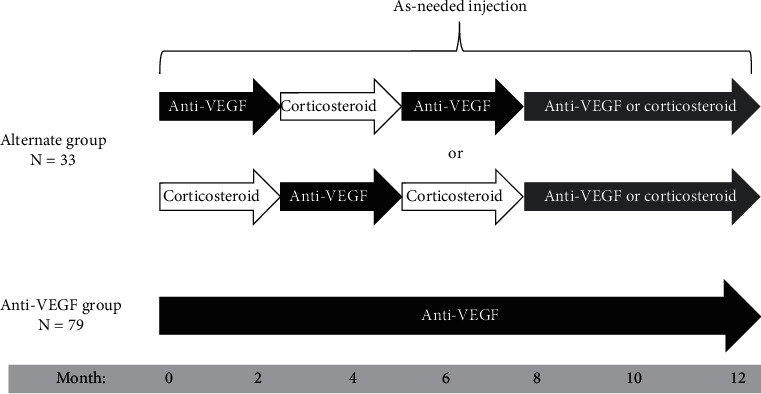
Schematic representation of the treatment sequence in the alternate group and the anti-vascular endothelial growth factor group.

Patients were classified into two groups according to the treatment received during the follow-up period: (1) alternate treatment with intravitreal corticosteroid and anti-VEGF injections (alternate group) and (2) treatment only with intravitreal anti-VEGF injections (anti-VEGF group). Patients in the alternate group were initially administered with anti-VEGF injections, followed by corticosteroid injections, and then switched back to anti-VEGF injections; alternatively, they were initially administered with corticosteroid injections, followed by anti-VEGF injections, and then switched back to corticosteroid injections. After the switch back, any medication was allowed.  Figure 1 shows the treatment scheme of the present study.

### 2.2. Outcome Measures

Outcome measures were assessed at 1, 2, 4, 8, 10, and 12 months. Primary outcome measures were mean changes from baseline in the BCVA and CMT at month 12. Secondary outcome measures were the number of total intravitreal injections administered during the 12-month follow-up. The safety outcome measures were the rate of development of cataract requiring surgical intervention, increase in IOP requiring additional medical or surgical intervention, the incidence of ocular adverse events including endophthalmitis and retinal detachment, and nonocular adverse events including myocardial infarctions and stroke in both groups during the follow-up period.

### 2.3. Statistical Analyses

The Snellen BCVA measurements were converted to logarithm of the minimum angle of resolution (logMAR) units to perform the statistical analysis. Data were expressed as either mean ± standard deviation or numbers and percentages. We compared the differences between the groups using a chi-square test or Fisher's exact test for categorical variables. Alternatively, paired *t*-test was used for comparing the related samples in each group, and Student's *t*-test for comparing between groups regarding continuous variables. In the subgroup analysis, propensity score matching was performed to compensate for the differences in the number of injections between the two groups. Statistical significance was attributed to a *P* value of <0.05. Statistical analyses were performed using IBM SPSS 22.0 (IBM SPSS Statistics for Windows, Version 22.0, IBM Corp., Armonk, NY).

## 3. Results

### 3.1. Basic Characteristics

In total, 112 eyes of 112 patients with ME secondary to RVO were included in the analysis. The mean age of the patients was 67.5 ± 11.2 years; 45 (40.2%) were male, and 67 (59.8%) were female. At baseline, 19 (17.0%) patients had diabetes. Sixty-eight (60.7%) eyes had ME secondary to branch retinal vein occlusion (BRVO), and 44 (39.3%) eyes had ME secondary to central retinal vein occlusion (CRVO). The mean BCVA was 0.82 ± 0.51 logMAR, and the mean CMT was 563.6 ± 239.7 *μ*m. Twenty (17.9%) eyes were pseudophakic. Mean IOP was 12.0 ± 3.5 mmHg. Two (1.8%) eyes had previously undergone focal laser treatment, and three (2.7%) eyes had received peripheral scatter laser treatment previously. The baseline characteristics of the alternate group (33 eyes) and the anti-VEGF group (79 eyes) are summarized in Table 1. There was no significant difference in sex, age, proportion of patients with diabetes, proportion of RVO type (BRVO or CRVO), BCVA, CMT, lens status, IOP, and prior treatment between the two groups.

### 3.2. Changes in Visual Acuity

**Figure 2:**
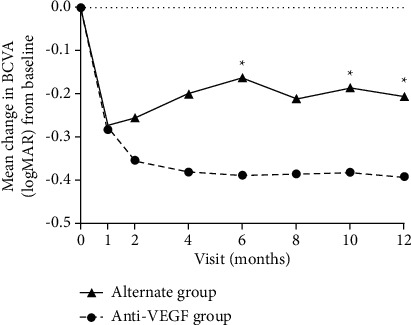
Mean change in best-corrected visual acuity in treatment-naïve eyes with macular edema eyes secondary to retinal vein occlusion that were alternately treated with intravitreal corticosteroid and anti-vascular endothelial growth factor (VEGF) injections (alternate group) or were treated only with intravitreal anti-VEGF injections (anti-VEGF group). ^*∗*^ indicate a statistically significant difference (*P* < 0.05) between two groups. BCVA = best-corrected visual acuity; logMAR = logarithm of the minimum angle of resolution.

Both groups showed significant increases in the mean BCVA after the first injection, and the improvement was sustained through 12 months with PRN injections (Figure 2). There were statistically significant intergroup differences in the mean change of BCVA values from the baseline to month 6 (*P*=0.045), month 10 (*P*=0.034), and month 12 (*P*=0.042) (Figure 2). At the end of the study, the alternate group demonstrated an average gain of 0.39 logMAR in BCVA, while the anti-VEGF group demonstrated an average gain of 0.21 logMAR.

### 3.3. Anatomical Changes

**Figure 3:**
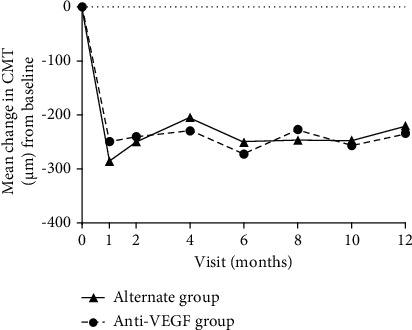
The mean change in the central macular thickness in treatment-naïve eyes with macular edema eyes secondary to retinal vein occlusion that were alternately treated with intravitreal corticosteroid and anti-vascular endothelial growth factor (VEGF) injections (alternate group) or were treated only with intravitreal anti-VEGF injections (anti-VEGF group). CMT = central macular thickness.

Both groups showed significant increases in mean CMT after the first injection, and the increase was sustained through 12 months with PRN injections (Figure 3). There were no statistically significant intergroup differences in the mean change in CMT at all time points. At the end of the study, the alternate group achieved an average reduction of 229.9 *μ*m in CMT, while the anti-VEGF group demonstrated an average reduction of 229.9 *μ*m (*P*=0.887). At month 12, ME was resolved in 16 (48.5%) eyes in the alternate group and in 37 (46.8%) eyes in the anti-VEGF group (*P*=0.309).

### 3.4. Variables and Safety Related to Treatment

The mean number of intravitreal injections during the 12-month study period was 5.2 (range 3–7) in the alternate group and 4.2 (range 3–9) in the anti-VEGF group (*P* < 0.001). The mean time to repeat injection was 3.1 months (range 1.2–6.1) in the alternate group and 2.9 months (range 0.9–6.5) in the anti-VEGF group (*P*=0.480).

**Figure 4:**
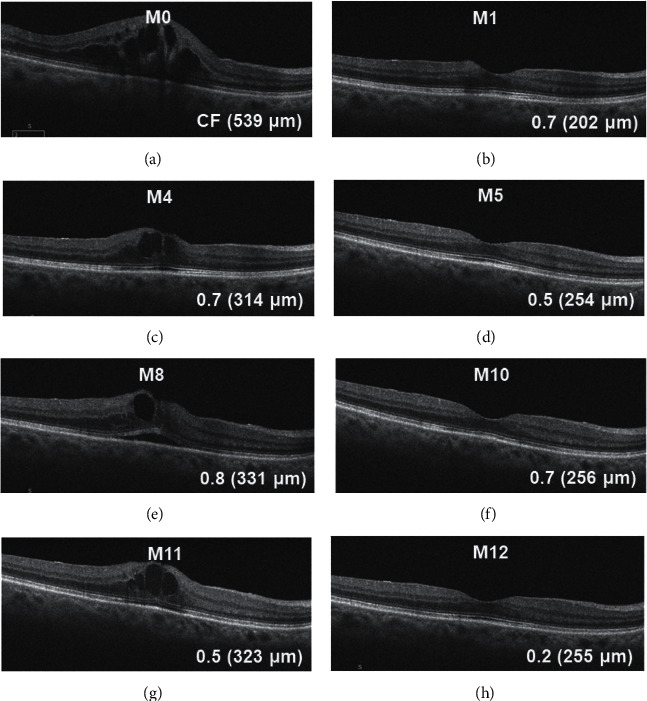
An 82-year-old woman with macular edema secondary to branch retinal vein occlusion alternately treated with intravitreal anti-vascular endothelial growth factor and corticosteroid. Serial changes on optical coherence tomography B scans, with visual acuity (VA, logMAR) and central macula thickness of the left eye. (A) Cystoid macular edema (CME) was noted. An intravitreal bevacizumab injection (IVB) was administered. (B) One month later, VA and CME had improved. IVB was repeated. (C) Three months later, an intravitreal sustained-release dexamethasone implant injection (IVD) was administered because of the aggravation of VA and CME. (D) Improvement in VA and CME was noted 1 month later. (E) Three months later, IVB was administered due to aggravation of VA and CME. (F) Two months later, VA and CME had improved. (G) One month later, IVD was administered depending on VA and CME aggravation. (H) One month later, VA and CME had improved. CF = counting finger.

In the alternate group, 27 eyes (81.8%) were initially treated with anti-VEGF agents and 6 (18.2%) eyes with corticosteroids. A mean of 2.7 (range 1–5) intravitreal anti-VEGF injections were administered before switching to corticosteroids, and a mean of 1.3 (range 1–2) intravitreal corticosteroid injections were administered before switching to anti-VEGF agents. Of the 172 injections in the alternate group, 49 (28.4%) were corticosteroids, and a mean of 1.5 (range 1–3) injections were administered to each patient.  Figure 4 shows a representative case.

Focal laser rescue was not performed in the alternate group, whereas it was performed in 4 (5.1%) eyes in the anti-VEGF group (*P*=0.575). Cataract surgery was performed in 6 (23.1%) of the 26 phakic eyes in the alternate group and in 3 (4.5%) of the 66 phakic eyes in the anti-VEGF group (*P*=0.014). 13 (39.4%) eyes in the alternate group and 16 (20.3%) eyes in the anti-VEGF group were pseudophakic at month 12 (*P*=0.035).

An increase in IOP required additional topical medication to reduce the IOP in two eyes each, in the alternate and anti-VEGF group (6.1% and 2.5%, respectively, *P*=0.580). No eye required additional procedures to normalize the IOP in the alternate group, while 1 eye (1.3%) in the anti-VEGF group required glaucoma surgery (*P*=1.000). There were no cases of significant complications, including endophthalmitis or retinal detachment, and no systemic serious adverse events including myocardial infarctions or stroke in both groups during the follow-up period.

### 3.5. Subgroup Analysis

A subgroup analysis evaluated outcomes among eyes that were phakic at month 12, for which changes due to the cataract surgeries would not affect the visual outcome. Among 83 phakic eyes at month 12, the mean change in BCVA from baseline to month 12 was 0.43 logMAR gain in the alternate group and 0.21 logMAR gain in the anti-VEGF group (*P*=0.045).

A one-to-one propensity score-matched cohort was created to compensate for the differences in the number of injections between the two groups. There were statistically significant intergroup differences in the mean change of BCVA values from the baseline to month 6 (*P*=0.029), month 8 (*P*=0.014), month 10 (*P*=0.024), and month 12 (*P*=0.048). At month 12, the alternate group demonstrated an average gain of 0.39 logMAR in BCVA, while the anti-VEGF group demonstrated an average gain of 0.17 logMAR (Figure S1).

## 4. Discussion

This study found that treatment with intravitreal corticosteroid and anti-VEGF injections alternately improved visual outcomes in patients with treatment-naïve ME secondary to RVO compared to intravitreal anti-VEGF monotherapy in a real-life setting. Both treatments resulted in similar favorable anatomical outcomes. However, the alternate treatment group required one additional injection than the intravitreal anti-VEGF monotherapy group during the 12-month treatment period. These results are in line with our previous study on DME, which showed more favorable visual outcomes in patients who received alternate treatment than anti-VEGF monotherapy [17]. To the best of our knowledge, this is possibly the first study that evaluated the efficacy of alternate treatment using intravitreal corticosteroid and anti-VEGF injections for ME secondary to RVO.

Inflammation plays a significant role in the development of ME secondary to RVO. Significantly high levels of inflammatory cytokines were noted in the vitreous or aqueous humor of ME secondary to RVO, resulting in the inflammatory breakdown of the blood-retinal barrier [15, 18]. Several intraocular inflammatory cytokines remain unchanged after anti-VEGF treatment [19, 20], which can play a significant role in the development of treatment resistance. Corticosteroids inhibit a large number of inflammatory molecules as well as VEGF, inhibit leukostasis, and stabilize and reconstitute the blood-retinal barrier [21, 22]. Considering a significant role of inflammation in the pathogenesis of ME secondary to RVO and persistent inflammatory cytokines after anti-VEGF treatment, the use of corticosteroid along with anti-VEGF, or the treatment switch to corticosteroid in patients with poor response to anti-VEGF, can be beneficial.

Some treatment strategies combining corticosteroid and anti-VEGF have been proposed, mainly involving simultaneous intravitreal injection of both medications or subsequent intravitreal corticosteroid injection at short intervals after intravitreal anti-VEGF injection [23]. However, these strategies might not be clinically feasible because of a higher risk of complications associated with increased puncture frequency or injection volume compared to anti-VEGF or corticosteroid monotherapy.

Several studies reported the results of switching from anti-VEGF agents to corticosteroids in suboptimal responders [24–26]. However, these studies continued only corticosteroids after switching and did not switch back to anti-VEGF agents, as in this study. The prolonged use of corticosteroids can also cause changes in the intraocular cytokine milieu and elevation of VEGF, which might result in treatment resistance. However, the alternate treatment can respond to changes in the cytokine milieu after the medication switch, as well as changes in VEGF and inflammatory cytokines, which might reduce treatment resistance and improve the treatment outcomes of ME secondary to RVO. This might be the theoretical basis for better results in the alternate group than in the anti-VEGF group in the present study.

In our study, both groups showed significant improvement in BCVA as early as 1 month after the initial injection. However, the anti-VEGF group demonstrated a gradual loss in BCVA from month 2. In contrast, the alternate group maintained the initial BCVA throughout the study period. As a result, the mean BCVA changes showed a significant difference between the two groups at months 6, 10, and 12. Although BCVA worsened from month 2 in the anti-VEGF group, the CMT did not show a corresponding increase. Previous studies reported relatively modest correlations between changes in visual acuity and CMT over time in DME eyes treated with intravitreal anti-VEGF injections [27]. However, the changes in the CMT could provide a clue. The decrease in BCVA at month 4 was in accordance with the increased CMT at month 4. The CMT of the anti-VEGF group was partially recovered at month 6 and maintained nearly stable until month 12; however, the decreased BCVA did not show recovery. This finding was consistent with that in the previous studies, which reported that ME could result in irreversible changes in the retinal layer, which leads to permanent deterioration of the visual function [28, 29]. Sun et al. [29] reported that the early 4-month change in the inner retinal layer disorganization caused by ME predicts visual acuity change from baseline to 1 year. Considering this, the worse visual outcome in the anti-VEGF group might be due to irreversible retinal layer changes in the early treatment period, mainly originating from the recurrence of ME.

The subgroup analysis of the propensity score-matched cohort compensated for the injection numbers produced similar results, a better visual outcome in the alternate group. This suggests that the better visual outcome in the alternate group might not have been due to more injections than the intravitreal anti-VEGF monotherapy group but from different treatment strategies, intravitreal anti-VEGF monotherapy versus alternate treatment with intravitreal corticosteroid and anti-VEGF injections.

The alternate group underwent more cataract surgeries during the follow-up period compared with the anti-VEGF group. Considering that the proportion of pseudophakic eyes showed no significant difference between the groups at baseline, more cataract surgeries during the follow-up period might have resulted from the significantly frequent cataract development due to corticosteroid use in the alternate group. However, the favorable visual outcome in the alternate group might not be due to the higher number of cataract surgeries because the subgroup analyses that included only eyes that were phakic at month 12 produced similar results.

The limitations to our study primarily stem from its retrospective design with a real-world setting. First, the treatment decisions could have differed between the treating physicians owing to the lack of a predefined treatment protocol. Reasons for changing the treatment strategy from the anti-VEGF monotherapy to the alternate treatment were not assessed. Second, the treatment medications used in this study varied. The subgroup analysis for each medication was not possible due to the limited number of subjects. Third, this study could not provide definitive evidence that alternate treatment with intravitreal corticosteroid and anti-VEGF is superior to intravitreal anti-VEGF monotherapy due to its retrospective study design and short-term period. However, this study provides evidence that alternate treatment with intravitreal corticosteroid and anti-VEGF can be a reasonable option for ME secondary to RVO, especially in pseudophakic eyes that are resistant to intravitreal anti-VEGF monotherapy. Fourth, as steroid-related cataract formation and/or progression is usually observed after 12 months, the developmental rate of cataracts requiring surgical intervention may be higher than the present study captured [30, 31]. Last, like many other studies [25, 32–37], we did not differentiate between BRVO and CRVO to extend the sample size. This may have biased the results considering the better natural history and better response to various treatment modalities of BRVO versus CRVO [31]. However, there was no statistically significant difference in the ratio of BRVO and CRVO between the alternate and anti-VEGF groups, which may have minimized the bias. Further large-scale prospective long-term studies with well-defined treatment protocols for CRVO and BRVO are necessary to confirm our results.

## 5. Conclusions

This study provides evidence that treatment with intravitreal corticosteroid and anti-VEGF injections alternately might result in better visual outcomes in patients with treatment-naïve ME secondary to RVO than in those undergoing intravitreal anti-VEGF monotherapy. This novel treatment strategy can enhance the overall treatment outcome of ME secondary to RVO, particularly in suboptimal responders to anti-VEGF treatment.

## Figures and Tables

**Table 1:** Baseline characteristics of the studied eyes.

	Alternate group (*n* = 33)	Anti-VEGF group (*n* = 79)	*P*
Males, *n* (%)	12 (36.4%)	33 (41.8%)	0.595^a^
Age, years (mean ± SD)	69.1 ± 11.5	66.8 ± 11.0	0.332^b^
Diabetes	8 (24.2%)	11 (13.9%)	0.185^a^
Retinal vein occlusion, *n* (%)			
BRVO	23 (69.7%)	45 (57.0%)	0.208^a^
CRVO	10 (30.3%)	34 (43.0%)	
Baseline BCVA (logMAR)	0.90 ± 0.58	0.80 ± 0.48	0.364^b^
Baseline CMT (*μ*m)	569.5 ± 258.5	561.1 ± 232.9	0.866^b^
Pseudophakic, *n* (%)	7 (21.2%)	13 (16.5%)	0.549^a^
IOP, mmHg	12.1 ± 3.4	11.9 ± 3.6	0.787^b^
Prior treatment, *n* (%)			
Focal laser	1 (3.0%)	1 (1.3%)	0.504^c^
Peripheral scatter laser	2 (6.1%)	1 (1.3%)	0.207^c^

BRVO, branch retinal vein occlusion; CRVO, central retinal vein occlusion; BCVA, best-corrected visual acuity; logMAR, logarithm of the minimum angle of resolution; CMT, central macular thickness; IOP, intraocular pressure. ^a^*P* values were calculated using the Chi-square test. ^b^*P* values were calculated using the Mann–Whitney *U*-test. ^c^*P* values were calculated using the Fisher's exact test.

## Data Availability

The data used to support the findings of this study are available from the corresponding author upon request.

## Supplementary Materials

Supplementary Materials *Supplement Figure 1*. Mean change in best-corrected visual acuity in 1 : 1 propensity score matched treatment-naïve macular edema eyes secondary to retinal vein occlusion that were alternately treated with intravitreal corticosteroid and anti-vascular endothelial growth factor (VEGF) injections (alternate group) or were treated only with intravitreal anti-VEGF monotherapy (anti-VEGF group) ^*∗*^ indicate a statistically significant difference (*P* < 0.05) between two groups. BCVA = best-corrected visual acuity; logMAR = logarithm of the minimum angle of resolution.
