# Supplementary material for: Effect of Alternate Treatment with Intravitreal Corticosteroid and Anti-VEGF for Macular Edema Secondary to Retinal Vein Occlusion
Source: J Ophthalmol. 2021 Sep 28;2021:5948113. doi: 10.1155/2021/5948113 (PMC8492286; doi:10.1155/2021/5948113)
Supplement: Supplementary Materials — Supplement Figure 1. Mean change in best-corrected visual acuity in 1 : 1 propensity score matched treatment-naïve macular edema eyes secondary to retinal vein occlusion that were alternately treated with intravitreal corticosteroid and anti-vascular endothelial growth factor (VEGF) injections (alternate group) or were treated only with intravitreal anti-VEGF monotherapy (anti-VEGF group) ∗ indicate a statistically significant difference (P < 0.05) between two groups. BCVA = best-corrected visual acuity; logMAR = logarithm of the minimum angle of resolution. [file 5948113.f1.docx]

**Supplement Figure 1:** Mean change in best-corrected visual acuity in 1:1 propensity score mathced treatment-naïve macular edema eyes secondary to retinal vein occlusion that were alternately treated with intravitreal corticosteroid and anti-vascular endothelial growth factor (VEGF) injections (alternate group) or were treated only with intravitreal anti-VEGF monotherapy (anti-VEGF group)





*Asterisks indicate a statistically significant difference (P < 0.05) between two groups.

BCVA = best-corrected visual acuity; logMAR = logarithm of the minimum angle of resolution
